# Deciphering the Role of Energy Transfer Configuration in PFAS Recognition by Metal‐Organic Frameworks

**DOI:** 10.1002/anie.6212058

**Published:** 2026-09-14

**Authors:** Jiatong Huo, Zongsu Han, Zhaoyi Liu, Xincheng Liu, Joshua Rushlow, Hong‐Cai Zhou

**Affiliations:** ^1^ Department of Chemistry Texas A&M University, College Station Texas USA; ^2^ Department of Chemistry The University of Sheffield, Western Bank Sheffield United Kingdom

**Keywords:** energy transfer configuration, lanthanide metal‐organic framework, PFAS detection, ratiometric sensing

## Abstract

Per‐ and polyfluoroalkyl substances (PFAS) have attracted widespread attention as persistent environmental contaminants. However, their rapid detection remains challenging due to their weak light absorption capacities. Luminescence sensing provides a rapid and efficient approach for PFAS monitoring, while ratiometric sensing can further amplify signal variations and improve detection sensitivity with self‐referenced optical responses. Lanthanide metal‐organic frameworks (Ln‐MOFs), featuring tunable porous environments, accessible host–guest interactions, and well‐defined multicolor emissions, have emerged as promising platforms for constructing ratiometric sensing materials. Nevertheless, current luminescence sensing has primarily focused on optimizing interactions between the framework and analytes, whereas the influence of intraframework energy transfer configuration on sensing performance remains largely unexplored. Here, Eu/Tb spatial organization is deliberately engineered in EuTb‐FDA to decipher the role of energy transfer configuration in PFAS recognition. By regulating the spatial distribution of emissive centers, the Tb^3+^‐to‐Eu^3+^ energy transfer pathway is precisely modulated, enabling distinct energy transfer behaviors. The optimized configuration couples PFAS‐induced host–guest interactions with directional lanthanide energy redistribution, resulting in enhanced sensing performance, thereby generating an amplified ratiometric response. This work reveals intraframework energy transfer configuration as a critical yet overlooked parameter in luminescence sensing and provides a new design principle for developing high‐performance ratiometric sensing materials.

## Graphical abstract

Spatial organization of Eu/Tb centers in EuTb‐FDA MOFs is employed to regulate Tb‐to‐Eu energy transfer configuration for ratiometric PFAS sensing. Directional energy transfer amplifies PFAS‐induced optical perturbations into opposite lanthanide emission responses, with enhanced Tb emission and quenched Eu emission, thereby amplifying the sensing efficiencies and providing guidance for the design of high‐performance luminescent sensing materials.

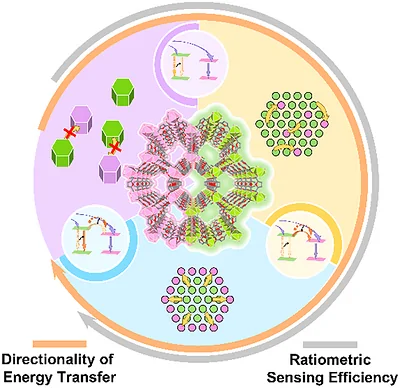

## 1 Introduction

Per‐ and polyfluoroalkyl substances (PFAS) have emerged as persistent environmental contaminants of global concern because their strong C─F bonds and amphiphilic structures endow them with exceptional chemical stability, mobility, and resistance to degradation [1, 2, 3]. Their widespread occurrence in natural ecosystems and bioaccumulation in living organisms have been associated with potential adverse health effects, including endocrine disruption, immune dysfunction, and increased risks of chronic diseases, highlighting the urgent need for rapid and reliable detection methods for timely monitoring and risk assessment. However, the rapid screening of PFAS remains challenging because most PFAS lack strong chromophores and intrinsic fluorophores, providing limited direct ultraviolet‐visible (UV‐vis) or fluorescence readouts [4, 5, 6]. Therefore, developing sensing pathways that can amplify weak PFAS‐induced perturbations into reliable and readable optical signals is important for practical PFAS monitoring [7, 8, 9, 10, 11, 12].

Ratiometric luminescent sensing offers a self‐calibrated strategy for improving the sensitivity and reliability of PFAS detection [13, 14]. By correlating the relative changes of different emission centers, this approach can effectively minimize systematic errors [15, 16, 17]. However, conventional sensing strategies have primarily focused on optimizing host–guest interactions between the framework and target analytes [18, 19], whereas the influence of intraframework energy transfer configuration on sensing performance remains largely unexplored. Such internal energy transfer processes may critically influence energy redistribution among emissive centers and consequently modulate sensing responses. Therefore, rational regulation of energy transfer configuration may provide an additional strategy for enhancing sensing responses and improving the performance of ratiometric luminescent sensing materials.

Metal‐organic frameworks (MOFs) provide an ideal model system for investigating these processes because their crystalline and modular structures enable the ordered organization of emissive components within well‐defined architectures [20, 21, 22, 23, 24]. Such structural regularity allows the spatial distribution of emissive centers to be systematically examined, thereby providing a basis for understanding the influence of energy transfer configuration [25, 26, 27, 28] on sensing behavior. Among MOFs, lanthanide‐based MOFs (Ln‐MOFs) are particularly attractive for ratiometric luminescent sensing because the similar coordination characteristics of lanthanide ions enable the facile incorporation of multiple emissive centers within a single framework, while their bright and characteristic emissions provide highly distinguishable optical signals for detecting subtle analyte‐induced variations [29, 30, 31]. Among various lanthanide pairs, Eu(III)/Tb(III) bimetallic systems have been widely studied because Eu(III) and Tb(III) exhibit bright and characteristic red and green emissions in the visible region, respectively [32, 33, 34, 35]. In these systems, the energy transfer process between Eu(III) and Tb(III) centers should influence the ratiometric sensing performance. However, the role of energy transfer configuration in regulating sensing responses has not yet been systematically investigated.

In this work, a mixed‐metal distribution strategy is applied to EuTb‐FDA MOFs (H_2_FDA = 2,5‐furandicarboxylic acid) to establish a spatially regulated energy transfer system for ratiometric PFAS sensing. The spatial distribution of Eu/Tb centers serves as a structural handle for regulating energy transfer mode and directionality, thereby shaping the resulting sensing efficiencies. Accordingly, three EuTb‐FDA samples were constructed and designated as RET‐EuTb‐FDA, DET‐EuTb‐FDA, and IET‐EuTb‐FDA, with RET, DET, and IET denoting random, directional, and inactive energy transfer, respectively (Scheme 1). These three samples serve as a comparative system for evaluating distinct energy transfer configurations through their PFAS sensing abilities. Among them, DET‐EuTb‐FDA exhibited the most effective ratiometric sensing behavior, converting PFAS‐induced perturbations into opposite changes in the two lanthanide emission channels, with decreased Eu^3+^ emission and increased Tb^3+^ emission. In contrast, RET‐EuTb‐FDA and IET‐EuTb‐FDA lacked this opposite dual‐channel modulation and showed only slight quenching or negligible changes in the Tb^3+^ emission channel, resulting in insensitive ratiometric responses. This comparison demonstrates that the intraframework energy transfer configuration is critical in sensing. The resulting structure–energy transfer‐response relationship identifies intraframework energy transfer configuration as a key structural parameter that links framework organization to external sensing performance, providing guidance for the rational design of sensitive sensing materials in the future.

**SCHEME 1:**
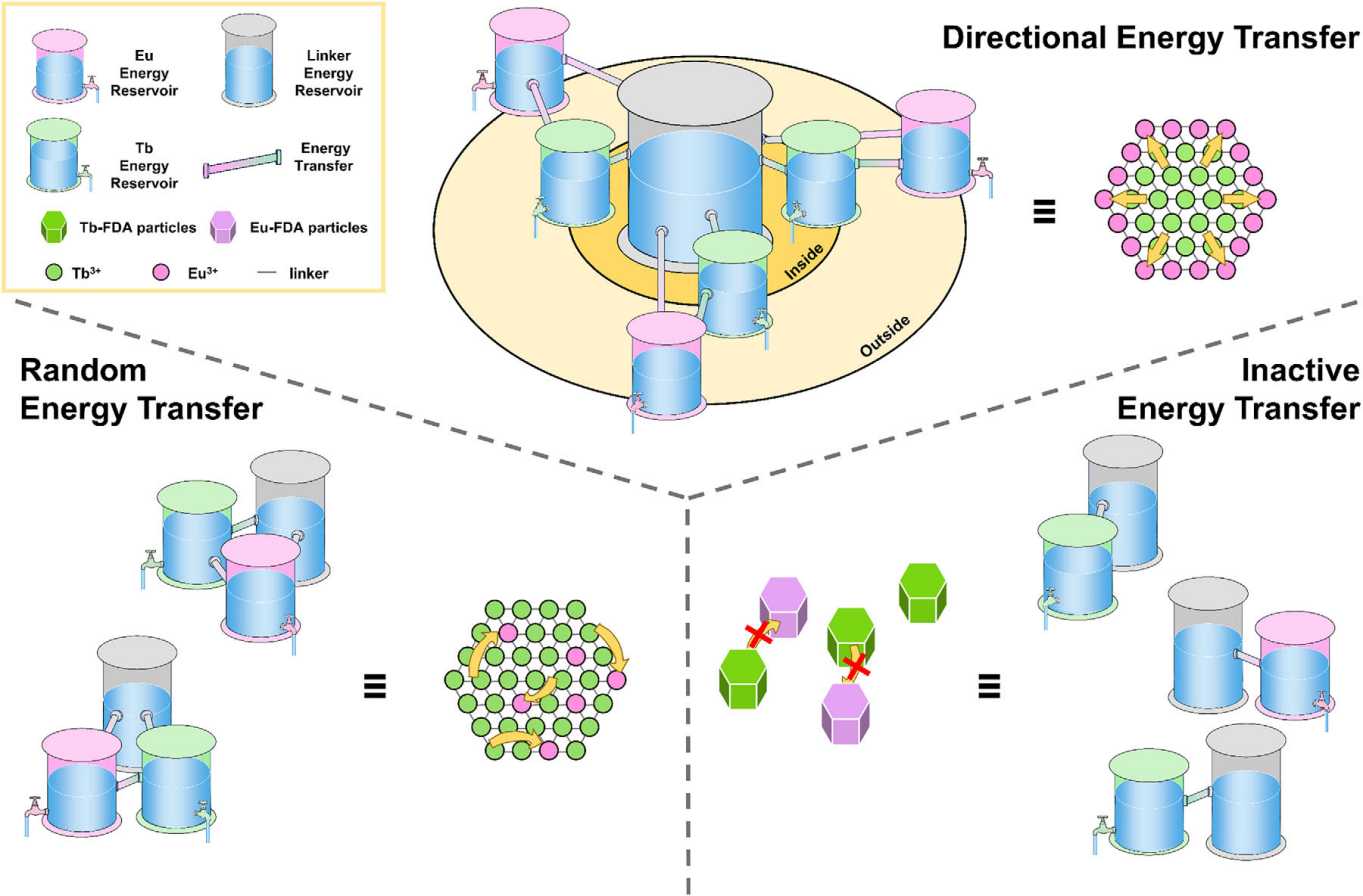
Schematic illustration of EuTb‐FDA MOFs with different spatial distributions of Eu and Tb centers, leading to random energy transfer (RET), directional energy transfer (DET), and inactive energy transfer (IET).

EuTb‐FDA was constructed using H_2_FDA as the organic linker and Eu/Tb ions as metal centers, forming a three‐dimensional framework constructed from seven‐coordinated Ln^3+^ centers and four‐connected FDA^2−^ linkers [36]. The Ln^3+^ ions are bridged by carboxylate groups to form one‐dimensional lanthanide chains, which are further connected by FDA^2−^ linkers into a porous framework with one‐dimensional honeycomb‐shaped channels along the [100] direction. In this system, the deprotonated FDA^2−^ linker not only supports framework formation but also harvests excitation energy and sensitizes lanthanide‐centered emissions through the “antenna effect” [36].

## 2 Materials and Methods

As illustrated in Figure 1, three EuTb‐FDA samples were designed to evaluate different energy transfer configuration pathways. RET‐EuTb‐FDA was prepared by one‐pot mixed‐metal synthesis, leading to random Tb^3+^‐to‐Eu^3+^ energy transfer within the framework in all directions. DET‐EuTb‐FDA was obtained through a two‐step process involving the initial synthesis of Tb‐FDA, followed by postsynthetic metal exchange to partially replace Tb^3+^ ions with Eu^3+^ ions, which generated an Eu‐rich exterior and Tb‐rich interior distribution and promoted directional energy transfer from inside (Tb^3+^) to outside (Eu^3+^). IET‐EuTb‐FDA was obtained by mechanically grinding a physical mixture of Eu‐FDA and Tb‐FDA particles, resulting in spatially separated Eu‐ and Tb‐emissive particles with inactive energy transfer between Eu^3+^ and Tb^3+^. Powder X‐ray diffraction (PXRD) patterns demonstrated that the obtained samples have similar structures as Eu‐FDA and Tb‐FDA, indicating successful framework formation and structural preservation after different mixed‐metal treatments (Figure S1). Infrared (IR) spectra showed that the C═O vibration of protonated carboxylic acid in free H_2_FDA at approximately 1680 cm^−1^ disappeared in EuTb‐FDA, while asymmetric and symmetric carboxylate stretching bands appeared at approximately 1574 and 1358 cm^−1^, supporting H_2_FDA deprotonation and FDA^2−^ coordination to lanthanide ions (Figure S2) [37]. Scanning electron microscopy (SEM) images showed the morphologies of RET‐EuTb‐FDA, DET‐EuTb‐FDA, and IET‐EuTb‐FDA (Figures S3–S5). Distinct Eu/Tb distribution modes among these three samples are confirmed by energy‐dispersive X‐ray spectroscopy (EDS) mapping: homogeneous Eu/Tb distribution in RET‐EuTb‐FDA, Eu‐rich exterior and Tb‐rich interior in DET‐EuTb‐FDA, and particle‐level separation of Eu‐FDA and Tb‐FDA components in IET‐EuTb‐FDA (Figures S6–S8). Inductively coupled plasma mass spectrometry (ICP‐MS) gave Tb/Eu molar ratios of 344:1 for RET‐EuTb‐FDA, 81:1 for DET‐EuTb‐FDA, and 13:1 for IET‐EuTb‐FDA (Figure S9). These Tb/Eu molar ratios were selected to achieve similar Tb at 542 nm and Eu at 613 nm emission intensity ratios in the emission spectra (Figure S10). This comparable initial ratiometric output provides a controlled basis for evaluating the effect of mixed‐metal distribution on sensing.

**FIGURE 1:**
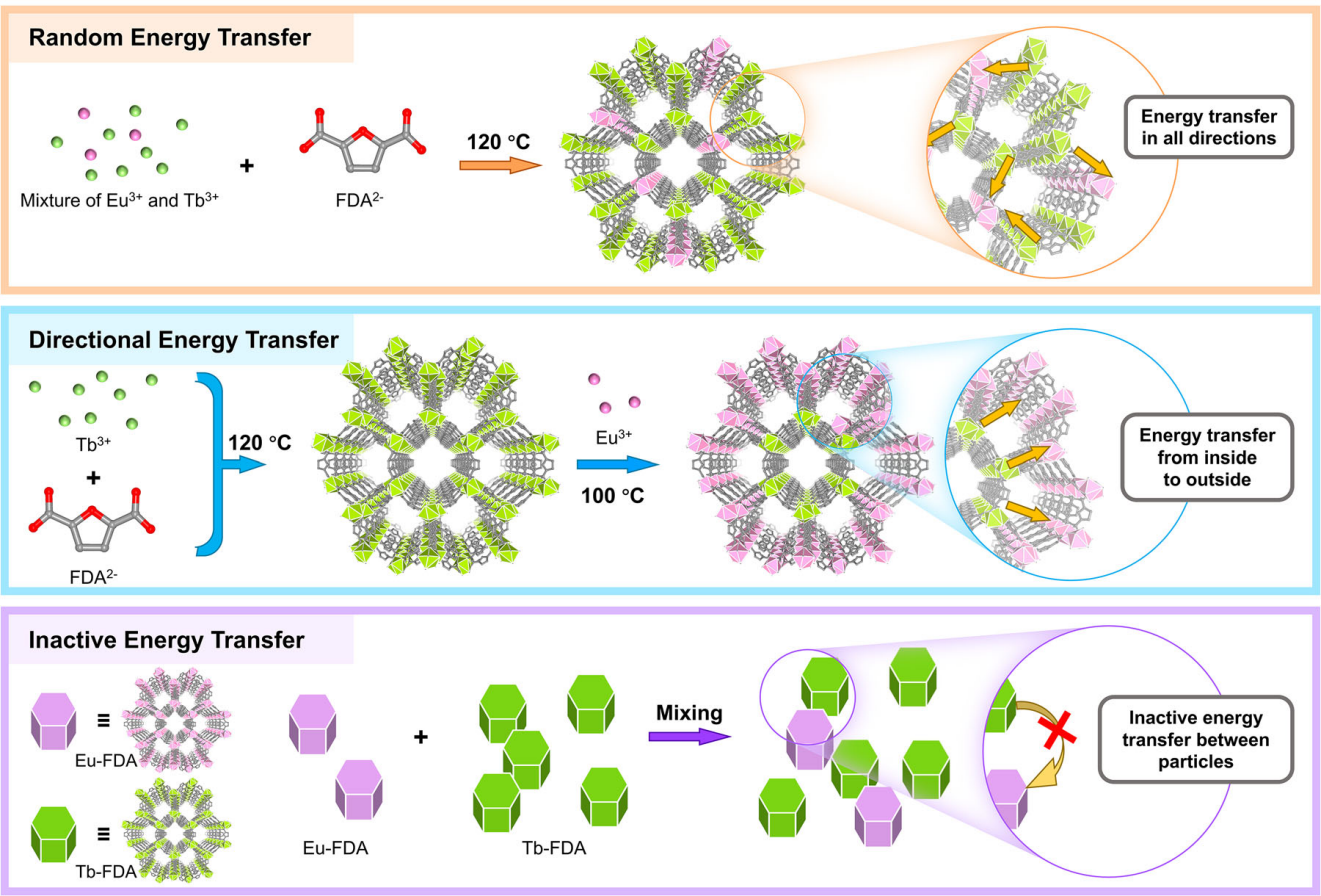
Construction of RET‐EuTb‐FDA, DET‐EuTb‐FDA, and IET‐EuTb‐FDA with Eu/Tb spatial distributions that generate random energy transfer, directional energy transfer, and inactive energy transfer. Atom code: C, gray; O, red; Tb, green; Eu, pink.

## 3 Result and Discussion

The luminescence stability of RET‐EuTb‐FDA, DET‐EuTb‐FDA, and IET‐EuTb‐FDA was first evaluated. All these sample suspensions maintained stable emission signals, indicating that the solvent environment did not cause significant luminescence fluctuation during the sensing measurements (Figures S11–S13). The Eu^3+^ emission at 613 nm and Tb^3+^ emission at 542 nm were selected for subsequent analysis because of their relatively high intensities and pronounced response changes. Upon the addition of perfluorobutanoic acid (PFBA), perfluorobutanesulfonic acid (PFBS), and perfluorononanoic acid (PFNA), the three samples showed distinct luminescence responses (Figures S14–S22). For all samples, the Eu^3+^ emission at 613 nm exhibited a similar decreasing trend with increasing analyte concentration. In contrast, the Tb^3+^ emission at 542 nm showed a clear dependence on the energy transfer configuration: it increased markedly in DET‐EuTb‐FDA, increased slightly in RET‐EuTb‐FDA, and decreased slightly in IET‐EuTb‐FDA (Figure 2a–c). The intensity changes at 542 nm and 613 nm were fitted with linear or exponential equations to describe their concentration‐dependent response toward different PFAS analytes (Figures S23–S31). Further, the ratiometric signal was analyzed by the intensity ratio between 542 and 613 nm, while DET‐EuTb‐FDA showed the largest ratio change among the three samples for PFBA, PFBS, and PFNA (Figure 2d–f), establishing directional energy transfer as the dominant amplification pathway for PFAS sensing. After the sensing measurements, PXRD patterns and IR spectra showed no obvious changes, supporting the retention of framework crystallinity and the overall coordination environment during PFAS detection (Figures S32–S49).

**FIGURE 2:**
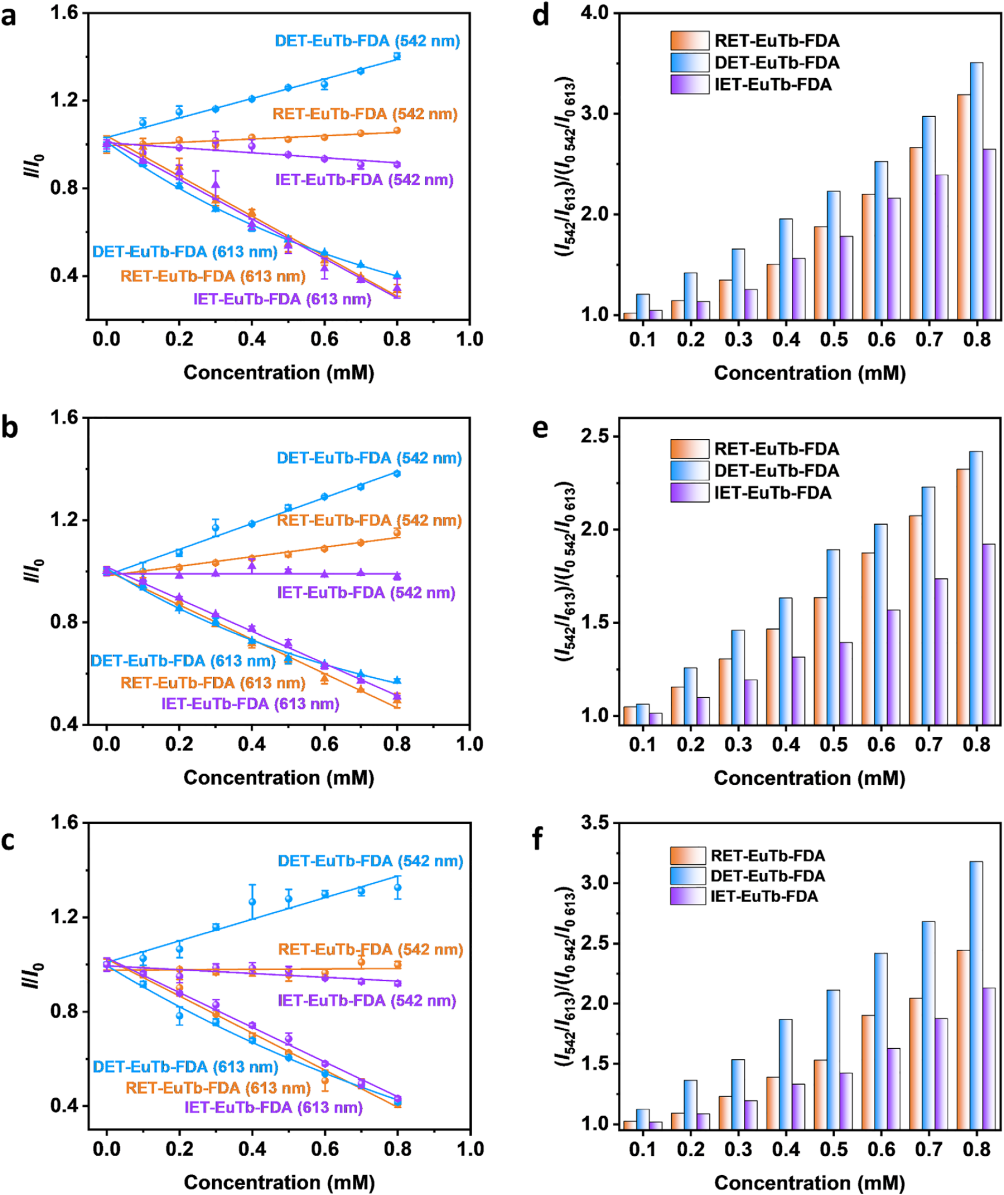
Luminescence responses of RET‐EuTb‐FDA, DET‐EuTb‐FDA, and IET‐EuTb‐FDA toward different PFAS analytes. Intensity changes with the addition of (a) PFBA, (b) PFBS, and (c) PFNA. Ratiometric responses based on the 542/613 nm emission intensity ratio toward (d) PFBA, (e) PFBS, and (f) PFNA.

To visually evaluate the differential responses of the two emission centers, the emission intensity changes at 613 nm and 542 nm in 0.8 mM analyte solutions are shown in Figure 3a, providing a channel‐level comparison in PFAS sensing among RET‐EuTb‐FDA, DET‐EuTb‐FDA, and IET‐EuTb‐FDA. DET‐EuTb‐FDA exhibited the strongest opposite modulation at the two selected emission wavelengths, with pronounced quenching at 613 nm accompanied by obvious enhancement at 542 nm. The Stern–Volmer quenching constants (*K*_SV_) for the quenched emission channels and the slope *K* values for the enhanced emission channels at 613 nm and 542 nm are shown in Figure 3b, further reflecting the distinct response behaviors under different energy transfer configuration pathways (Figures S50–S52). Figure 3c shows that RET‐EuTb‐FDA produced a slightly higher ratiometric response than IET‐EuTb‐FDA, while DET‐EuTb‐FDA generated a much larger ratio change than RET‐EuTb‐FDA. Figure 3d further demonstrates that this directional pathway provides the steepest slope within the linear response range, giving DET‐EuTb‐FDA the best ratiometric sensitivity. Time‐dependent luminescence measurements showed that the emission signals reached a stable state within 1 min after analyte addition, demonstrating the rapid sensing capability of these samples (Figures S53–S61).

**FIGURE 3:**
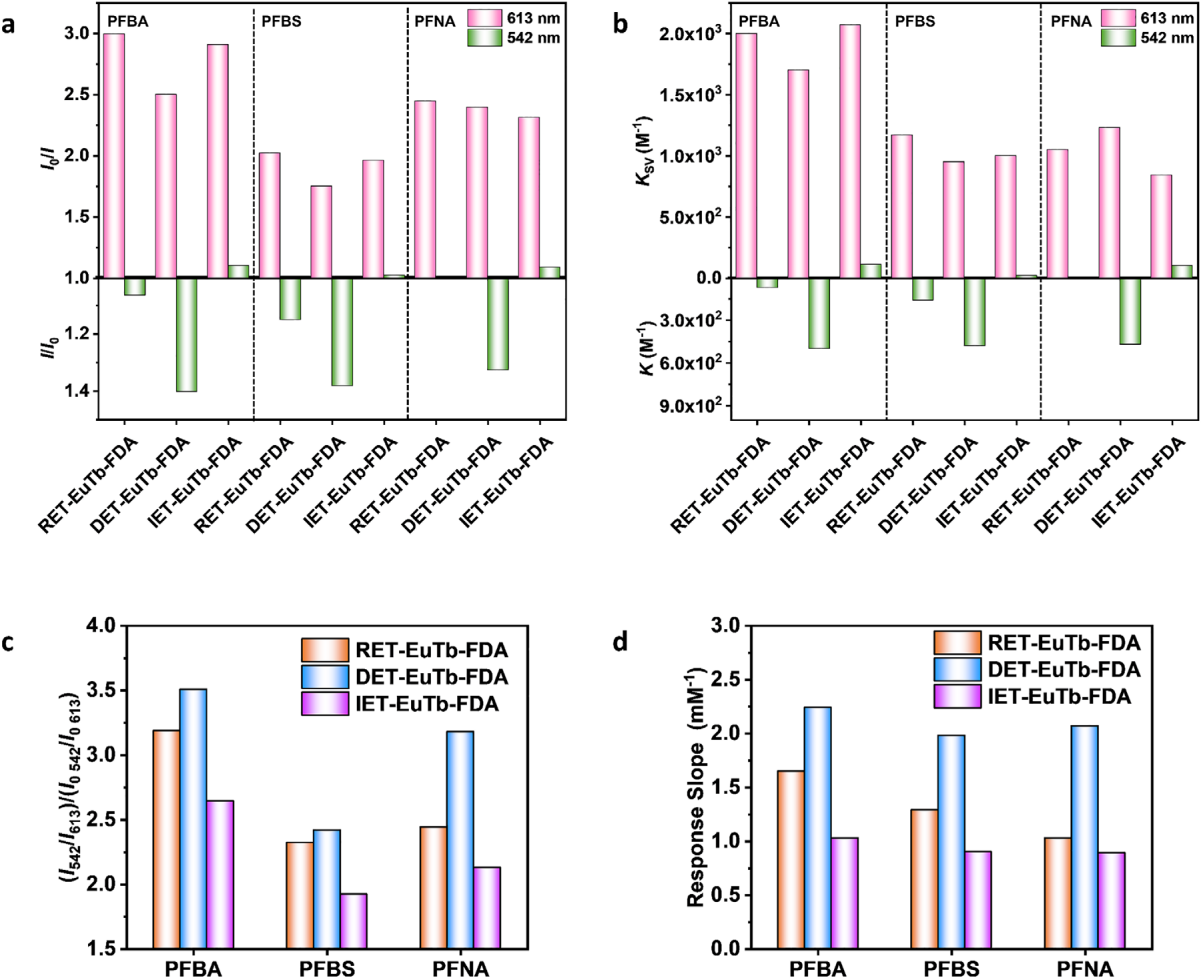
Luminescence responses of RET‐EuTb‐FDA, DET‐EuTb‐FDA, and IET‐EuTb‐FDA toward different PFAS analytes. (a) Luminescence intensity variations after the addition of 0.8 mM PFBA, PFBS, and PFNA. (b) Corresponding *K*_SV_ and the slope *K* values obtained from the concentration‐dependent fitting at 613 nm and 542 nm. (c) Ratiometric intensity variations after the addition of 0.8 mM PFBA, PFBS, and PFNA. (d) Ratiometric response slopes determined by fitting the linear regions of the concentration‐dependent response curves.

Mechanisms were systematically investigated including the luminescence and response mechanism. First, the luminescence mechanism of the monometallic Ln‐FDA MOFs was studied in detail. For the monometallic MOFs, the luminescence mechanism is primarily governed by the ligand‐to‐metal energy transfer process. The singlet excited state was estimated from the UV‐vis absorption spectrum (Figure S62), while the triplet excited state was calculated through the phosphorescence spectrum at 77 K [38] of isostructural Gd‐FDA (Figure S63). Gd‐FDA was selected because the lowest excited state of Gd^3+^ (^6^P_7/2_) lies above the ligand triplet state, making ligand‐to‐Gd^3+^ energy transfer energetically unfavorable and allowing ligand‐centered phosphorescence to be observed at 77 K. The resulting spectrum therefore provides an estimate of the ligand triplet‐state energy, which can be compared with the emitting levels of Eu^3+^ and Tb^3+^ to assess the energetic feasibility of ligand‐to‐metal energy transfer [38]. In addition, the triplet‐state energy of the ligand is well matched with the excited energy levels of Eu^3+^ or Tb^3+^ to enable ligand‐to‐metal energy transfer. The proposed energy transfer pathway is summarized in Figure S64. Upon excitation, energy is first promoted from the ground state of the ligand to its singlet excited state, followed by intersystem crossing to the triplet excited state [39]. Subsequently, part of the triplet‐state energy is transferred to the excited states of Eu^3+^ or Tb^3+^ through ligand‐to‐metal energy transfer, followed by radiative relaxation to the ground states, giving rise to the characteristic lanthanide emissions [40].

Then, the luminescence mechanism of the mixed‐metal systems was analyzed. To facilitate mechanistic discussion, the ligand‐to‐Tb^3+^, ligand‐to‐Eu^3+^, and Tb^3+^‐to‐Eu^3+^ energy transfer processes are denoted as pathways **I**, **II**, and **III**. In IET‐EuTb‐FDA, Eu‐FDA, and Tb‐FDA are present as physically separated particles. Therefore, the ligand independently sensitizes Tb‐ and Eu‐centered emissions through the “antenna effect” via pathways **I** and **II**, whereas Tb^3+^‐to‐Eu^3+^ energy transfer through pathway **III** is negligible (Figure 4a). In RET‐EuTb‐FDA, the relatively homogeneous Eu/Tb distribution within the same particle enables not only the ligand‐to‐metal energy transfer pathways **I** and **II**, but also the metal‐to‐metal energy transfer pathway **III** (Figure 4b). In DET‐EuTb‐FDA, all of the energy transfer pathways are present. In addition, the Eu‐rich exterior and Tb‐rich interior create a spatially biased Tb^3+^‐to‐Eu^3+^ energy transfer pathway, thereby imparting directionality to pathway **III** (Figure 4c). Luminescence lifetime analysis further quantified the Tb^3+^‐to‐Eu^3+^ energy‐transfer efficiencies, which were calculated to be 75.60% for RET‐EuTb‐FDA, 32.98% for DET‐EuTb‐FDA, and 13.48% for IET‐EuTb‐FDA (Figures S65–S68).

**FIGURE 4:**
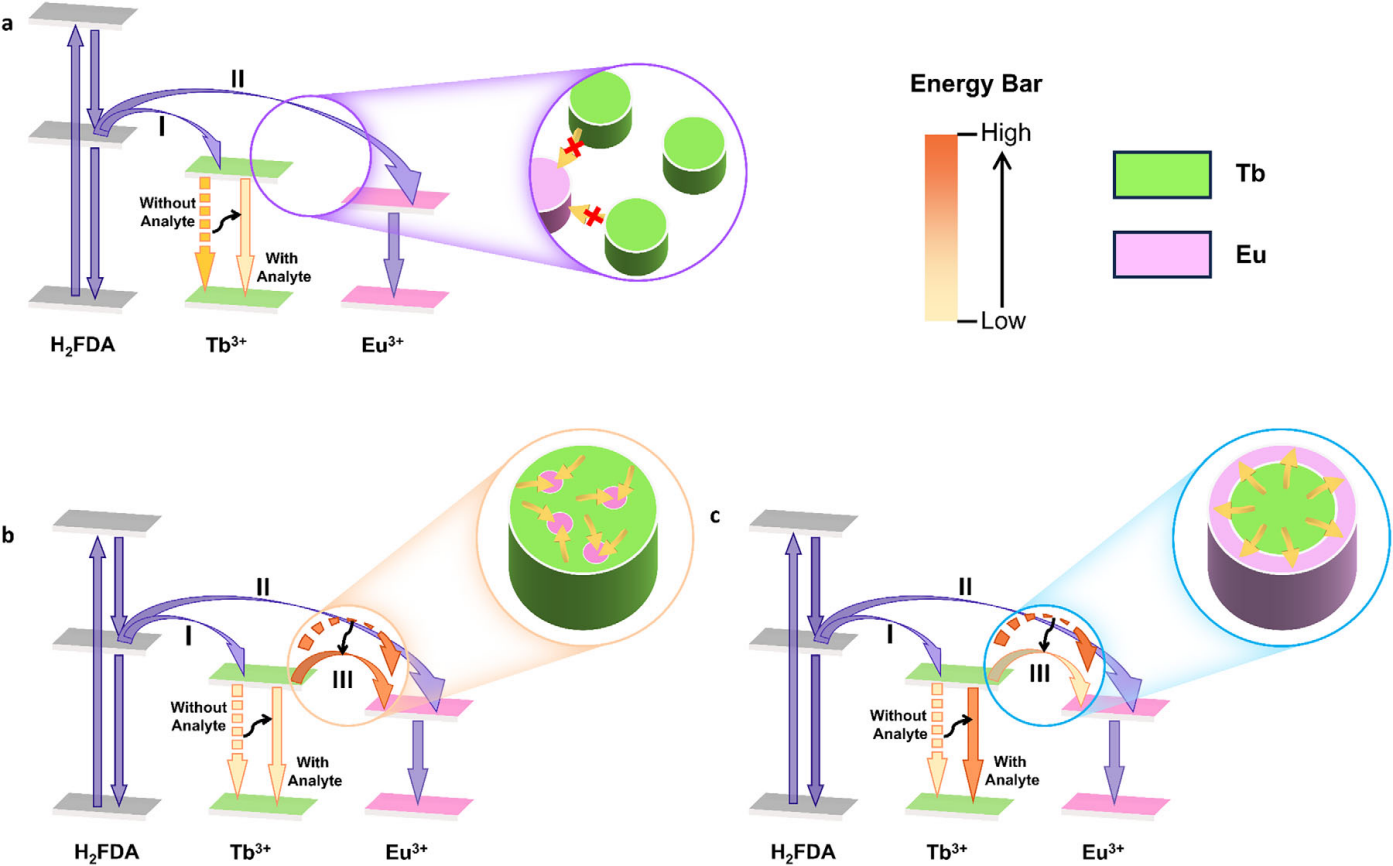
Proposed energy transfer pathways in EuTb‐FDA samples with different Eu/Tb spatial distributions. (a) Independent ligand‐to‐lanthanide sensitization in IET‐EuTb‐FDA with inactive interparticle energy transfer. (b) Coupled ligand sensitization and Tb^3+^‐to‐Eu^3+^ energy transfer in RET‐EuTb‐FDA. (c) PFAS‐perturbed Tb^3+^‐to‐Eu^3+^ energy transfer in DET‐EuTb‐FDA, where weakened Tb^3+^‐to‐Eu^3+^ energy transfer enhances Tb^3+^ emission.

The response mechanisms were analyzed from energy transfer mode and directionality by comparing the three EuTb‐FDA systems. For the energy transfer mode, the influence of pathway **III** was first evaluated by comparing IET‐EuTb‐FDA with RET‐EuTb‐FDA/DET‐EuTb‐FDA. In IET‐EuTb‐FDA, pathway **III** is negligible because Eu‐FDA and Tb‐FDA exist as physically separated particles (Figure 4). Therefore, PFAS‐induced perturbation mainly weakens the independent ligand‐to‐metal sensitization pathways **I** and **II**, leading to simultaneous decreases in both Eu^3+^ and Tb^3+^ emissions. In contrast, the presence of additional Tb^3+^‐to‐Eu^3+^ energy transfer through pathway **III** in RET‐EuTb‐FDA/DET‐EuTb‐FDA introduces an extra energy transfer channel for the system. Upon PFAS addition, the quenching of Eu^3+^ emission suggests that both pathway **II** and pathway **III** are suppressed. The suppression of pathway **III** partially compensates Tb‐centered emission by decreasing Tb^3+^‐to‐Eu^3+^ energy loss, thereby resulting in a slight enhancement of Tb^3+^ emission.

For the energy transfer directionality, the influence was further elucidated by comparing RET‐EuTb‐FDA and DET‐EuTb‐FDA (Figure 4b,c). In RET‐EuTb‐FDA, the relatively homogeneous Eu/Tb distribution leads to a nondirectional pathway **III**, allowing PFAS analytes to perturb both pathway **I** and pathway **III**. As a result, the suppression of pathway **III** partially offsets the weakening of pathway **I**, leading to a slight enhancement of Tb^3+^ emission. In contrast, DET‐EuTb‐FDA possesses a Eu‐rich exterior and Tb‐rich interior, which establishes a spatially biased and directional pathway **III**. Upon PFAS addition, the analytes mainly interact with the outer Eu‐rich region and strongly suppress pathway **III**, whereas pathway **I** sensitization in the inner Tb‐rich region is less affected. Consequently, the reduction of pathway **III** loss dominates over the weakening of pathway **I** sensitization, allowing more Tb^3+^ excited states to undergo radiative decay and producing the pronounced emission enhancement of Tb^3+^ emission observed for DET‐EuTb‐FDA. This comparison demonstrates that spatial organization not only determines the presence of metal‐to‐metal energy transfer but also controls its perturbation direction and extent, thereby regulating the final ratiometric sensing response.

## 4 Conclusion

In summary, this work demonstrates that the PFAS sensing performance of mixed‐lanthanide MOFs can be effectively regulated through intraframework energy transfer configuration. Comparison among RET‐, DET‐, and IET‐EuTb‐FDA reveals that metal‐to‐metal energy transfer plays a decisive role in sensing performance, while both its mode and directionality critically determine the recognition efficiency. The existence of energy transfer establishes an excitation energy reservoir that can accumulate and redistribute excitation energy, whereas its directionality determines the site at which PFAS‐induced optical perturbations are introduced into the energy transfer pathways, thereby governing the response mode and sensing efficiency. This study highlights the importance of intraframework energy transfer configuration in PFAS sensing, which establishes a fundamental relationship among metal spatial distribution, energy transfer directionality, and sensing response, providing guidance for the rational design of high‐performance luminescent sensing materials.

## Author Contributions

**Jiatong Huo**: conceptualization, writing – original draft, writing – review and editing, methodology, validation, investigation, visualization. **Zongsu Han**: conceptualization, writing – review and editing, methodology. **Zhaoyi Liu**: writing – review and editing. **Xincheng Liu**: validation. **Joshua Rushlow**: writing – review and editing. **Hong‐Cai Zhou**: writing – review and editing.

## Conflicts of Interest

The authors declare no conflicts of interest

## Supporting information




**Supporting File**: anie74799‐sup‐0001‐SuppMat.docx.

## Data Availability

The data that support the findings of this study are available from the corresponding author upon reasonable request.
