# Supplementary material for: Deciphering the Role of Energy Transfer Configuration in PFAS Recognition by Metal‐Organic Frameworks
Source: Angew Chem Int Ed Engl. 2026 Sep 14;65(43):e6212058. doi: 10.1002/anie.6212058 (PMC13676422; doi:10.1002/anie.6212058)
Supplement: Supplementary file 1 [file ANIE-65-e6212058-s001.docx]

**Supporting Information**

**Deciphering the Role of Energy Transfer Configuration in PFAS Recognition by Metal-Organic Frameworks**

Jiatong Huo,^[a]^ Zongsu Han,*^,[a]^ Zhaoyi Liu,^[a]^ Xincheng Liu,^[b]^ Joshua Rushlow,^[a]^ and Hong-Cai Zhou*^,[a]^

[a] Department of Chemistry, Texas A&M University, College Station, Texas 77843, United States

E-mail: zongsu-han@tamu.edu; zhou@chem.tamu.edu

[b] Department of Chemistry, The University of Sheffield, Western Bank, Sheffield S10 2TN, United Kingdom

**Table of Content**

Materials and Methods	2

Basic Characterizations	4

Luminescence Sensing	9

Sensing Mechanisms	35

Reference	39

**Materials and Methods**

**Materials**

All reagents were commercially available and used without further purification. PXRD measurements were performed using a Bruker Powder-ECO X-ray diffractometer with Cu-Kα radiation. IR spectra were tested by a Shimadzu IRAffinity-1S spectrometer. Luminescence spectra were recorded on a Horiba Fluoromax-4 fluorescence spectrophotometer and a Shimadzu RF-5301 fluorescence spectrophotometer. Luminescence lifetime measurements were performed using an FLS1000 photoluminescence spectrometer (Edinburgh Instruments Ltd., Livingston, UK) equipped with a microsecond flashlamp (μF). UV-vis absorption spectra were measured by a Shimadzu UV-2450 absorption spectrometer. ICP-MS measurements were performed using a PerkinElmer NexION 300D ICP-MS instrument. SEM and EDS were collected using an FEI Quanta 600 FE-SEM instrument.

**Synthesis**

**Tb-FDA:** Tb-FDA was synthesized in reference to the literature.^1^ Tb(NO_3_)_3_·6H_2_O (0.1 mmol, 45 mg), H_2_FDA (0.2 mmol, 31 mg) and DMF 2 mL were added in a 4 mL sealed glass bottle under ultrasound and then heated at 120 ℃ for one day. Then the crystals were collected by filtration, washed with fresh DMF, and dried in air.

**Eu-FDA:** Eu-FDA was synthesized in reference to the literature.^1^ Eu(NO_3_)_3_·6H_2_O (0.1 mmol, 45 mg), H_2_FDA (0.2 mmol, 31 mg) and DMF 2 mL were added in a 4 mL sealed glass bottle under ultrasound and then heated at 120 ℃ for one day. Then the crystals were collected by filtration, washed with fresh DMF, and dried in air.

**RET-EuTb-FDA**: A mixture of Tb(NO_3_)_3_·6H_2_O (0.1 mmol, 45 mg), 0.01 M Eu(NO_3_)_3_·6H_2_O DMF solution 0.029 mL (Eu/Tb molar ratio ~ 1:345), H_2_FDA (0.2 mmol, 31 mg) and DMF 2 mL were added in a 4 mL sealed glass bottle under ultrasound and then heated at 120 ℃ for one day. Then the crystals were collected by filtration, washed with fresh DMF, and dried in air.

**DET-EuTb-FDA**: Tb-FDA crystals were soaked in a 0.1 M Eu(NO_3_)_3_ DMF solution at 100 °C for 2 days, yielding a Eu/Tb-FDA sample with comparable emission intensities at 542 and 613 nm. The crystals were then collected by filtration and washed with fresh DMF.

**IET-EuTb-FDA**: This sample was prepared by thoroughly mixing and grinding Tb-FDA (25 mg) and Eu-FDA (2 mg), yielding a mixture with comparable emission intensities at 542 and 613 nm.

**Luminescence Experiments**

All samples were ground into fine powder before use. The samples for luminescence experiments were dispersed in DMF by ultrasound for 10 minutes to form a clear suspension with a concentration of 0.3 mg mL^-1^. Each line was tested after 10 seconds of ultrasound treatment.

**Pre-treatment process for EDS mapping of DET-EuTb-FDA**

The DET-EuTb-FDA sample was embedded in epoxy resin to prepare a cross-section for EDS mapping. LX-112 embedding epoxy and Araldite epoxy resin were mixed with dodecenyl succinic anhydride as the hardener. The MOF crystals were placed into Beem 00 specimen embedding capsules, and the mixed epoxy resin was added to fill the capsules. The samples were then cured in an oven at 50 °C overnight. After curing, the hardened epoxy blocks containing the embedded MOF crystals were removed from the capsules and faced off to expose a flat cross-section of the sample. The cross sections were prepared using a Leica EM UC7 ultramicrotome equipped with a freshly cut 6.4 mm glass knife, which was made using a Leica EM KMR 3 knifemaker.

**Basic Characterizations**

**Figure S1.** PXRD patterns of RET-EuTb-FDA, DET-EuTb-FDA, IET-EuTb-FDA, Eu-FDA, Tb-FDA and the simulated sample.

**Figure S2.** IR spectra of RET-EuTb-FDA, DET-EuTb-FDA, IET-EuTb-FDA, Eu-FDA, Tb-FDA and H_2_FDA.

**Figure S3.** SEM image of the RET-EuTb-FDA.

**Figure S4.** SEM image of the DET-EuTb-FDA.

**Figure S5.** SEM image of the IET-EuTb-FDA.

**Figure S6.** EDS mapping of the RET-EuTb-FDA.

**Figure S7.** Cross-section EDS mapping of the DET-EuTb-FDA.

**Figure S8.** EDS mapping of the IET-EuTb-FDA.

**Figure S9.** Tb/Eu molar ratios of the RET-EuTb-FDA, DET-EuTb-FDA, and IET-EuTb-FDA MOFs determined by ICP-MS.

**Luminescence Sensing**

**Figure S10.** Emission spectra of the RET-EuTb-FDA, DET-EuTb-FDA, and IET-EuTb-FDA.

**Figure S11.** Emission spectra and intensity changes at 542 and 613 nm of RET-EuTb-FDA with time.

**Figure S12.** Emission spectra and intensity changes at 542 and 613 nm of DET-EuTb-FDA with time.

**Figure S13.** Emission spectra and intensity changes at 542 and 613 nm of IET-EuTb-FDA with time.

**Figure S14.** Emission spectra of RET-EuTb-FDA with the additions of PFBA.

**Figure S15.** Emission spectra of RET-EuTb-FDA with the additions of PFBS.

**Figure S16.** Emission spectra of RET-EuTb-FDA with the additions of PFNA.

**Figure S17.** Emission spectra of DET-EuTb-FDA with the additions of PFBA.

**Figure S18.** Emission spectra of DET-EuTb-FDA with the additions of PFBS.

**Figure S19.** Emission spectra of DET-EuTb-FDA with the additions of PFNA.

**Figure S20.** Emission spectra of IET-EuTb-FDA with the additions of PFBA.

**Figure S21.** Emission spectra of IET-EuTb-FDA with the additions of PFBS.

**Figure S22.** Emission spectra of IET-EuTb-FDA with the additions of PFNA.

**Figure S23.** Intensity changes and the fitting curves for RET-EuTb-FDA with the additions of PFBA.

**Figure S24.** Intensity changes and the fitting curves for RET-EuTb-FDA with the additions of PFBS.

**Figure S25.** Intensity changes and the fitting curves for RET-EuTb-FDA with the additions of PFNA.

**Figure S26.** Intensity changes and the fitting curves for DET-EuTb-FDA with the additions of PFBA.

**Figure S27.** Intensity changes and the fitting curves for DET-EuTb-FDA with the additions of PFBS.

**Figure S28.** Intensity changes and the fitting curves for DET-EuTb-FDA with the additions of PFNA.

**Figure S29.** Intensity changes and the fitting curves for IET-EuTb-FDA with the additions of PFBA.

**Figure S30.** Intensity changes and the fitting curves for IET-EuTb-FDA with the additions of PFBS.

**Figure S31.** Intensity changes and the fitting curves for IET-EuTb-FDA with the additions of PFNA.

**Figure S32.** IR spectra of RET-EuTb-FDA before and after PFBA sensing.

**Figure S33.** IR spectra of RET-EuTb-FDA before and after PFBS sensing.

**Figure S34.** IR spectra of RET-EuTb-FDA before and after PFNA sensing.

**Figure S35.** IR spectra of DET-EuTb-FDA before and after PFBA sensing.

**Figure S36.** IR spectra of DET-EuTb-FDA before and after PFBS sensing.

**Figure S37.** IR spectra of DET-EuTb-FDA before and after PFNA sensing.

**Figure S38.** IR spectra of IET-EuTb-FDA before and after PFBA sensing.

**Figure S39.** IR spectra of IET-EuTb-FDA before and after PFBS sensing.

**Figure S40.** IR spectra of IET-EuTb-FDA before and after PFNA sensing.

**Figure S41.** PXRD patterns of RET-EuTb-FDA before and after PFBA sensing.

**Figure S42.** PXRD patterns of RET-EuTb-FDA before and after PFBS sensing.

**Figure S43.** PXRD patterns of RET-EuTb-FDA before and after PFNA sensing.

**Figure S44.** PXRD patterns of DET-EuTb-FDA before and after PFBA sensing.

**Figure S45.** PXRD patterns of DET-EuTb-FDA before and after PFBS sensing.

**Figure S46.** PXRD patterns of DET-EuTb-FDA before and after PFNA sensing.

**Figure S47.** PXRD patterns of IET-EuTb-FDA before and after PFBA sensing.

**Figure S48.** PXRD patterns of IET-EuTb-FDA before and after PFBS sensing.

**Figure S49.** PXRD patterns of IET-EuTb-FDA before and after PFNA sensing.

**Figure S50.** Emission response slope *k* of RET-EuTb-FDA, DET-EuTb-FDA, and IET-EuTb-FDA toward PFBA.

**Figure S51.** Emission response slope *k* of RET-EuTb-FDA, DET-EuTb-FDA, and IET-EuTb-FDA toward PFBS.

**Figure S52.** Emission response slope *k* of RET-EuTb-FDA, DET-EuTb-FDA, and IET-EuTb-FDA toward PFNA.

**Figure S53.** Emission spectra and intensity changes of RET-EuTb-FDA with the additions of PFBA with time.

**Figure S54.** Emission spectra and intensity changes of RET-EuTb-FDA with the additions of PFBS with time.

**Figure S55.** Emission spectra and intensity changes of RET-EuTb-FDA with the additions of PFNA with time.

**Figure S56.** Emission spectra and intensity changes of DET-EuTb-FDA with the additions of PFBA with time.

**Figure S57.** Emission spectra and intensity changes of DET-EuTb-FDA with the additions of PFBS with time.

**Figure S58.** Emission spectra and intensity changes of DET-EuTb-FDA with the additions of PFNA with time.

**Figure S59.** Emission spectra and intensity changes of IET-EuTb-FDA with the additions of PFBA with time.

**Figure S60.** Emission spectra and intensity changes of IET-EuTb-FDA with the additions of PFBS with time.

**Figure S61.** Emission spectra and intensity changes of IET-EuTb-FDA with the additions of PFNA with time.

**Sensing Mechanisms**

**Figure S62.** UV-vis absorption spectrum of H_2_FDA.

**Figure S63.** Phosphorescence spectrum of Gd-FDA at 77 K.

**Figure S64.** Proposed energy-transfer diagram in EuTb-FDA.

**Figure S65.** Time-resolved luminescence decay profile of RET-EuTb-FDA monitored at 542 nm of Tb^3+^. The orange points and red curve represent the experimental data and fitted decay curve, respectively.

**Figure S66.** Time-resolved luminescence decay profile of DET-EuTb-FDA monitored at 542 nm of Tb^3+^. The blue points and red curve represent the experimental data and fitted decay curve, respectively.

**Figure S67.** Time-resolved luminescence decay profile of IET-EuTb-FDA monitored at 542 nm of Tb^3+^. The violet points and red curve represent the experimental data and fitted decay curve, respectively.

**Figure S68.** Time-resolved luminescence decay profile of Tb-FDA monitored at 542 nm of Tb^3+^. The black points and red curve represent the experimental data and fitted decay curve, respectively.

**Reference**

[1] J. Zhou, H. Li, H. Zhang, H. Li, W. Shi, P. Cheng, “A bimetallic lanthanide metal-organic material as a self-calibrating color-gradient luminescent sensor”, Advanced Materials **27**, (2015): 7072-7077.
